# Large-Scale Genomic Analysis of Codon Usage in Dengue Virus and Evaluation of Its Phylogenetic Dependence

**DOI:** 10.1155/2014/851425

**Published:** 2014-07-17

**Authors:** Edgar E. Lara-Ramírez, Ma Isabel Salazar, María de Jesús López-López, Juan Santiago Salas-Benito, Alejandro Sánchez-Varela, Xianwu Guo

**Affiliations:** ^1^Laboratory of Molecular Biomedicine, Center of Biotechnology on Genomics, National Polytechnic Institute, Colonia Narciso Mendoza, 88710 Reynosa, TAMPS, Mexico; ^2^Laboratory for Cellular Immunology and Immunopathogenesis, Department of Immunology, National School for Biological Sciences (ENCB), National Polytechnic Institute, 11340 New Mexico, DF, Mexico; ^3^Laboratory for Biomedicine, Department of Virology, National School of Medicine and Homeopathy, National Polytechnic Institute, 11340 New Mexico, DF, Mexico

## Abstract

The increasing number of dengue virus (DENV) genome sequences available allows identifying the contributing factors to DENV evolution. In the present study, the codon usage in serotypes 1–4 (DENV1–4) has been explored for 3047 sequenced genomes using different statistics methods. The correlation analysis of total GC content (GC) with GC content at the three nucleotide positions of codons (GC1, GC2, and GC3) as well as the effective number of codons (ENC, ENCp) versus GC3 plots revealed mutational bias and purifying selection pressures as the major forces influencing the codon usage, but with distinct pressure on specific nucleotide position in the codon. The correspondence analysis (CA) and clustering analysis on relative synonymous codon usage (RSCU) within each serotype showed similar clustering patterns to the phylogenetic analysis of nucleotide sequences for DENV1–4. These clustering patterns are strongly related to the virus geographic origin. The phylogenetic dependence analysis also suggests that stabilizing selection acts on the codon usage bias. Our analysis of a large scale reveals new feature on DENV genomic evolution.

## 1. Introduction

Dengue virus (DENV) is a positive strand RNA virus that belongs to the Flaviviridae family [1]. Its genome is approximately 11 kb long with an uninterrupted open reading frame (ORF) that encodes a polyprotein. DENV commonly exists as four (DENV1–4) distinct but genetically related serotypes. A new serotype (DENV5) has been recently described [2]. DENV exists in either sylvatic or human transmission cycles [3], which are most prevalent in tropical and subtropical areas, where ecoepidemiologic conditions contribute to sustaining the virus in nature. According to the World Health Organization, ≈2.5 billion people living in >100 countries are at risk of being infected by one or more of the DENV serotypes [4]. DENV are the cause of dengue fever and the more complicated forms of diseases, dengue haemorrhagic fever and dengue shock syndrome. At the present, there is no effective vaccine to prevent dengue diseases and no drug for specific therapy.

The degeneracy is an intrinsic characteristic of genetic code and enables different codons to encode for a given amino acid. However, the choice of synonymous codons is not random for a species; therefore, codon usage varies among species [5]. Some factors seem to influence the codon usage; for example, mutational bias has been attributed as the major determinant of codon usage variation among RNA viruses [6]. In addition, the codon usage deviations are the evolutionary consequence of an organism [5] and the result of adaptive interaction between pathogenic viruses and their hosts [7]. Thus, it has been proposed that codon usage is useful to discern the evolutionary relationships between species [8] and the patterns of codon variation may also shed some light on fundamental questions on basic biology.

The analyses of codon usage in DENV have been previously studied in the context of genus* flavivirus* [7, 9, 10], RNA type viruses [6, 11], or DENV genomic comparisons [12–15]. These studies have provided some valuable information; however, only a limited number of genomes were employed for their analysis. The increasing number of genome sequences reported from all over the world could thus help to reveal how DENV genomes diverge and what the principal contributing factors for their evolution are. Here, the genome-wide codon usage patterns were analyzed for 3047 full-length genomes of DENV1–4. In addition, we applied two methods to assess the phylogenetic dependence of codon usage to unravel novel evolutionary features of DENV.

## 2. Materials and Methods

### 2.1. Genome Sequences

The whole genome sequences of 3047 DENV1–4 were downloaded from the NCBI DENV resource at http://www.ncbi.nlm.nih.gov/genomes/VirusVariation/Database/nph-select.cgi?taxid=12637 This website provided DENV information that includes sample sequence, location, and serotype [16]. Four datasets that correspond to each one of the four serotypes were established. They included 1336 genomes for DENV1, 927 genomes for DENV2, 670 genomes for DENV3, and 114 genomes for DENV4. The coding sequences of genomes were collected in a dataset for each serotype orderly according to their geographic regions of isolation as Africa, Asia, North America, Oceania, and South America and for the samples from the same continent along with the order of host sources as human, mosquito, monkey, and unknown host. A number was then assigned to each genome in each dataset, which facilitates the subsequent analyses. The accession numbers as well as the assigned numbers for corresponding genomes in the present study are provided in an excel spreadsheet in the Supplementary Material available online at http://dx.doi.org/10.1155/2014/851425.

### 2.2. Nucleotide Compositions and Codon Usage Bias

The total GC% (GC) and GC% at the 1st (GC1), 2nd (GC2), and 3rd (GC3) codon positions of coding sequences for each DENV genome sequence were calculated in order to show the impact of selection on codon usage of DENV. The total GC content was calculated with the following equation:

(1) $$\begin{array}{c} \text{GC}=\frac{\left(\text{G}+\text{C}\right)}{\left(\text{A}+\text{T}+\text{G}+\text{C}\right)}, \end{array}$$

where the G, C, A, and T are the number of nucleotides in the genome. For the calculation of GC at the three codon positions we used the following equation:

(2) $$\begin{array}{c} \text{GC}n=\frac{\text{G}n+\text{C}n}{\left(L/3\right)}, \end{array}$$

where G*n*, C*n* are the number of guanines and cytosines at the *n*th (1, 2, or 3) position of the codon and *L* is the length of the genome.

Relative synonymous codon usage (RSCU) [17] was estimated as a proportion of the observed occurrence of codons to the expected occurrence when all codons for the same amino acid are equally used. The RSCU was calculated with the following equation:

(3) $$\begin{array}{c} \text{RSCU}=\frac{X_{ij}}{\sum_{j}^{ni}X_{ij}}n_{i}, \end{array}$$

where *X*_*ij*_ is the observed number of the *i*th codon for the *j*th amino acid which has *n*_*i*_ kinds of synonymous codons. It was measured for 59 codons except Met, Trp, and the three stop codons for each genome tested in this study. Effective number of codons (ENC) is a parameter to reveal the number of equally used codons that could yield the observed codon usage bias in a gene or a genome [18]. ENC was calculated with the following equation:

(4) $$\begin{array}{c} \text{ENC}=2+\frac{9}{\overset{-}{F}2}+\frac{1}{\overset{-}{F}3}+\frac{5}{\overset{-}{F}4}+\frac{3}{\overset{-}{F}6}, \end{array}$$

where $\overset{-}{F}k$ (*k* = 2,3, 4,6) is the mean of $\overset{-}{F}k$ values for the *k*-fold degenerate amino acids. ENC's values range from 20, the strongest bias, to 61, no bias. Because the genomes of DENV have unique uninterrupted polyprotein ORF, we applied ENC to quantify the level of codon usage bias on genome level in the present study. ENC prime (ENCp) was also used to quantify the codon bias taking into account the nucleotide background of the genomes [19]. The GC at three codon positions and RSCU were calculated with package seqinr [20] for R [21], and ENC and ENCp were calculated with the software Codonw and the software ENC prime, respectively [19, 22].

### 2.3. Correspondence Analysis

Correspondence analysis (CA) is an effective method to show the relationship among multiple categorical variables by a statistical procedure. The unique condition is to have a nonnegative data ordered in a two-way table for analysis. It is much better if the table consists of large enough dataset and homogenous variables [23]. Our RSCU dataset forms a table that should meet well the CA conditions. The RSCU table was read and formatted as data.frame in order to perform the CA with the function “*dudi.coa*” using the ADE-4 package *⁠*[24] in R. In the results obtained, each genome was represented as 59-orthogonal axes, and each axis corresponds to one of 59 codons. Thus, the results of CA show how much DENV genomes are correlated to the level of codon usage variation patterns. The advantage of CA is that the results can be depicted as a map, in which each row and each column are represented as a point, which facilitates the understanding of the relation of codon usage bias among the genomes.

### 2.4. Evaluation of Influencing Evolutionary Factors of DENV Codon Usage

Correlation analysis of GC1, GC2, GC3, GC, ENC, and ENCp values and the selected axis of variation of each DENV1–4 dataset was performed, using Pearson's rank correlation method. For better explanation of the correlation results, only the coefficient ≥0.70 was considered as strong correlation [25, 26]. As regards the evaluation of correlation coefficients, the null hypothesis of no correlation between the variables was tested at significance level of *P* = 0.01.

### 2.5. Hierarchical Clustering Based on Codon Usage

A distance matrix that accounts for differences in RSCUs for DENV genomes was constructed with the function “*dist*” and the Euclidean distance method by the software R. The matrix obtained was then used to aggregate the RSCU values of each genome sequence into hierarchical clusters of similar codon patterns with the function “*hclust*” and the Ward method by the software R. The* hclust* objects produced were then transformed to phylo objects for plotting the final trees with the ape [27] and pyloch packages for R.

### 2.6. Alignments, Phylogenetic Trees, and Recombination Analysis

A phylogenetic analysis was also performed, based on the nucleotide sequences of genomes, to compare the result with that of clustering analysis based on the codon usage. The software MAFFT was used to align the whole DENV genomes of coding regions [28]. We used the default “*—auto*” function to run the alignments on MAFFT. The FastTree [29] software was used to construct approximately maximum-likelihood phylogenetic trees for each of DENV1–4 from alignments data. FastTree software can handle large alignments in a practical amount of time and memory. The generalized time-reversible (GTR) model was used for phylogenetic tree construction. To estimate the local support values of each split in the tree, the Shimodaira-Hasegawa test was used. The* Newick* tree files generated were used with the ape and pyloch packages for R to plot the phylogenetic trees. As the recombination has also impact on the evolution of DENV [30], we also tested this pattern using the software Recombination Analysis Tool (RAT) [31] for each DENV dataset.

### 2.7. Evaluation of Phylogenetic Dependence of Codon Usage

Phylogenetic dependence is a frequently employed test to evaluate the correlation of phenotypical traits with phylogenetic tree [32]. Such analysis was recently applied for codon usage bias in mosquitos [33]. In our study, two measures [34] (Abouheif's Cmean and Blomberg's *K*) have been applied to evaluate the dependence of codon usage values with the inferred phylogenetic tree of DENV1–4. To estimate Abouheif's Cmean, we firstly constructed phylo4d objects which contain the combined DENV1–4 phylogeny and the RSCU data.frame and then created a matrix of phylogenetic proximities between the tips of inferred phylogeny for each DENV1–4 dataset with the function “*proxTips*” and the method oriAbouheif. Finally, the function “*abouheif.moran*” and the method oriAbouheif were applied for the DENV1–4 phylo4d objects and DENV1–4 proximity phylogenetic matrix to calculate Abouheif's Cmean. The package adephylo [35], containing the functions “*proxTips*” and “*abouheif.moran,*” and the package phylobase containing the phylo4d constructors are both for R. To perform Blomberg's *K* test, we employed the function “*multiPhylosignal”* of the package picante for R [36]. This function allows the calculation of phylogenetic dependence for the RSCU DENV1–4 data.frame. We firstly resolved multifurcations (nodes of the tree with two or more descending branches) of the inferred DENV1–4 phylogenetic trees with branches of zero lengths using the function “*multi2di*” of the package ape [27]. In the tests of Abouheif's Cmean and Blomberg's *K*, the observed codon values for each DENV1–4 dataset were randomly permutated through the tips of each DENV1–4 tree and calculated the focal indices on the new, randomized codon pattern. The repetition of the process for 999 times produced a distribution of the focal indices under random codon usage variation. In comparison of the observed values with these random codon distributions, we took out the quantiles from the tested indices. The quantiles superior than 0.95 for significance level of 0.05 were considered [34].

## 3. Results

### 3.1. The G+C Content and ENC

**Figure 1:**
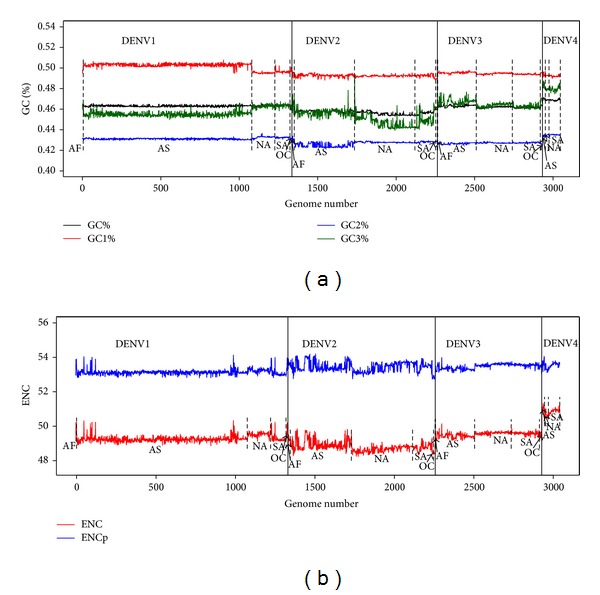
The nucleotide composition (G+C) and ENC, ENCp for the 3047 DENV1–4 genomes tested. (a) Total GC and GC content at the three codon positions for each genome. (b) ENC and ENCp for each genome. The dashed lines in both figures indicate the geographical separation within a DENV serotype. The abbreviations mean the following: AF, Africa; AS, Asia; NA, North America; SA, South America; OC, Oceania.

The overall G+C patterns at three nucleotide positions of codons were distinct for each DENV serotype (Figure 1(a), Table S1). The percentage of GC at the first nucleotide position of codon, GC1, is always the highest and that of GC2 is the lowest. GC1 in Asia was the highest in comparison to the other regions. The total GC showed variability among the serotypes (Figure 1(a)) and a characteristic pattern was observed for each serotype. GC3 was more variable than GC1 or GC2 in general and its change was in expense of GC content at preceding positions, particularly GC2. The GC3 value was very close to the total GC and also showed high relationship to it (Table S2). DENV4 had higher GC3 than other serotypes. Meanwhile, the variation profile in GC content among genomes within a DENV serotype was apparently related to their geographic origin (Figure 1(a)).

A matrix correlation analysis with the total GC content and the GC at the three nucleotide positions of codons is shown in Table S2. The total GC content showed a strong correlation with the GC3 (*r* ≥ 0.7, *P* = 0.01) for DENV1–4. GC1 had also a strong correlation with GC2 and GC3 in DENV1.

**Figure 2:**
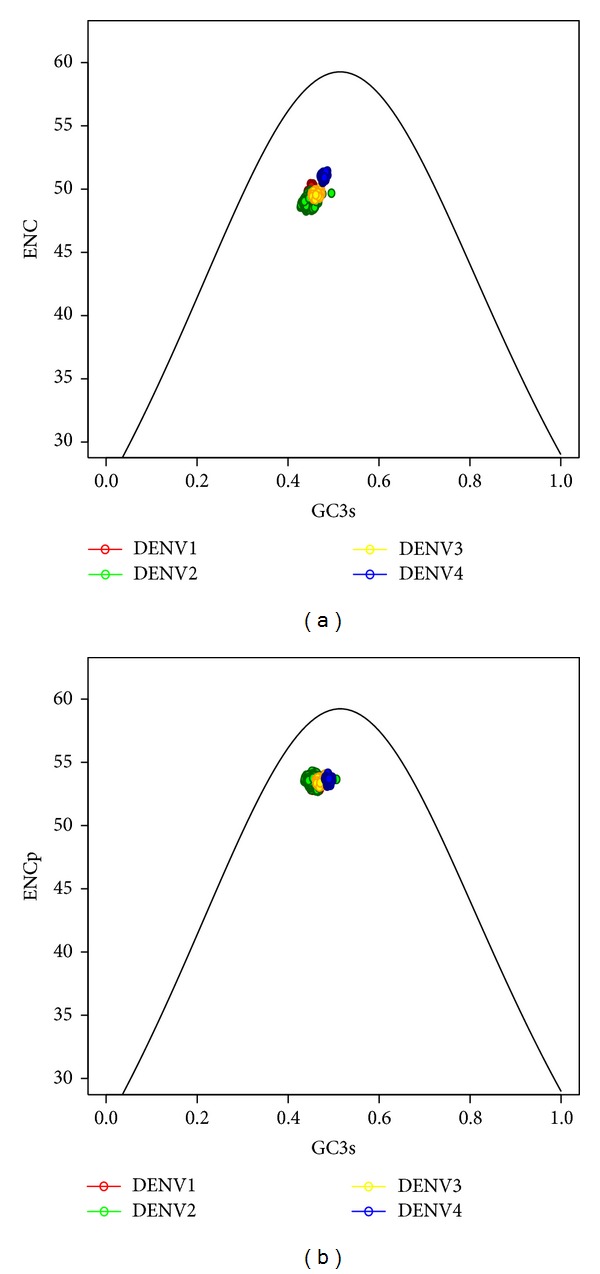
Effective number of codons versus GC3s plot of genomes of DENV1–4. (a) ENC versus GC3s, (b) ENCp versus GC3s.

The ENC was also analyzed for each serotype (Table S1). DENV2 appeared with the highest codon bias with a mean 48.8 ± 0.28 whereas DENV4 showed the lowest bias mean (50.87 ± 0.17). ENC bias among genomes within a DENV serotype was correlated with their geographic origin (Figure 1(b), red line). The ENCp analysis showed that the four serotypes had a homogenous codon bias in contrast to ENC (Figure 1(b), blue line). A curve of ENC and ENCp values for each DENV1–4 genome versus their corresponding GC3s data is shown in Figures 2(a) and 2(b). All points of the genome coding sequences lay below the predictable curve. The correlation analysis of ENC and ENCp showed almost no correlation with GC at any of the three codon positions for all DENV1–4 (Table S2). These results indicate that, independent of compositional constraint, some other factors that affect the codon usage variations exist.

### 3.2. Preferred Codons

The mean and standard deviation of RSCU for 18 amino acids except Met, Trp, and stop codons were determined for each serotype (Table S3). Eleven preferred codons, AGA(Arg), AAC(Asn), GAC(Asp), GAA(Glu), GGA(Gly), ATA(Ile), AAA(Lys), CCA(Pro), TCA(Ser), ACA(Thr), and GTG(Val) were consistently shared for all the four DENV (highlighted in blue). There was no extreme bias in preferred codons among specific serotypes. Although in some cases we observed the preferred codons for specific serotypes, these codons still belonged to the set of codons mainly used for the other serotypes. For example, DENV1 used more commonly TAT(Tyr) codon instead of the preferred TAC(Tyr) by DENV2–4. The CAC(His), not CAT(His), codon was preferred in DENV1 and DENV3 while DENV2 and DENV4 use these two codons Tyr and His at proximate frequency. Codon CTG(Leu) was preferred by DENV1 and DENV2, but DENV3 and DENV4 preferred the codon TTG(Leu).

### 3.3. Correspondence Analysis

One factorial axis accounted for 41.8%, 39.6%, and 40.9% of the total variability in DENV1–3, respectively, indicating that one factor was predominant for those serotypes while for DENV4 dataset the first axis accounted for 25%. The first two axes accounted for more than half of that variability (53–56%) for DENV1–3 except for DENV4 (41%). Thus, the first two factorial axes contribute to the principal differences in codon usage for DENV datasets.

**Figure 3:**
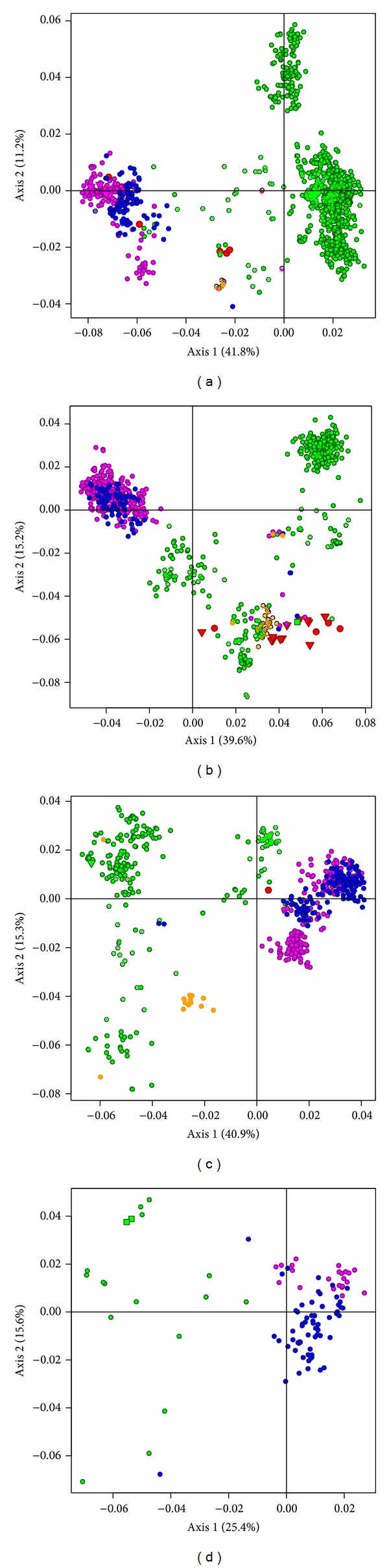
Correspondence analysis based on RSCU values for DENV. The geographic regions of isolates are indicated in colors as red (African), green (Asian), magenta (North American), blue (South American), and orange (Oceanic). The host sources are, respectively, represented as circles for human, squares for monkey, inverted triangles for mosquito, and asterisks for unknown host. (a) DENV1; (b) DENV2; (c) DENV3; (d) DENV4.

The factor maps produced by crossing axes with the major sources of variation showed well-demarcated geographic separation. They exhibit the following features: (1) in the first axis, as the most important factor on the maps for each serotype (Figures 3(a)–3(d)), the genomes were divided into clusters according to their geographic origin; (2) the Asian, African, and Oceanic genome sequences tend to cluster together; (3) the North American and South American genomes clustered together; (4) the Asian genomes appeared more dispersed than those from other regions; (5) the genomes from other hosts (mosquito, monkey, and unknown host) also clustered accordantly with their geographic sites of isolation. DENV2 showed the most complex geographic pattern. On the other hand, there were also some noticeable “outliers” in the figure, that is, the genomes that were not located in the cluster with the majority of strains sharing the same geographic origin (Figure 3, Table S4). Some of these strains have been previously mentioned outside the general cluster in their phylogenetic analysis. For example, the genome from Djibouti, Africa, of serotype 1, previously observed more closely related to the Asian strains due to the existence of a recombinant sequence region with a strain from Singapore (Asia) [37], was located on the Asian cluster in our analysis. Based on this finding we tested if the other identified outliers are recombinant strains with the software RAT. However, no sign of recombination was detected.

The correlations of the GC, GC1, GC2, GC3, ENC, and ENCp of each genome with its position on the first axis are shown in Table 1. Depending on the specific serotype, the genome position on the first axis had strong correlation with GC1 and GC3 for DENV1 and with GC2 and GC3 for DENV2 while others showed less correlation. It is interesting that GC3 showed negative relation with the first axis of the major variation for DENV1 but showed positive correlation with DENV2. ENC and ENCp showed no important correlation with all DENV1–4.

### 3.4. Phylogenetic and Hierarchical Clustering-Based Trees

The Hierarchical Clustering-Based Trees (HCbT) resulting from the RSCU data are shown in Figures S1(a)–(d). The HCbT revealed two major clusters in each serotype virus. The clusters consisting of Asian, African, and Oceanic genome sequences tend to group together, whereas the clusters enclosing South and North American sequences assemble together. However, some Asian genomes were located at the clusters of North and South American strains. The genomes identified as outliers were also located in the same geographical clusters as indicated in our CA analysis. On the other hand, the phylogenetic relationships among DENV genomes were also constructed based on the genome nucleotide sequences (Figures S1(e)–(h)). The comparison of these phylogenetic trees showed that these analyses on two datasets showed similar results. Moreover, the majority of the outliers were also confirmed by the inferred phylogenetic trees.

### 3.5. Evaluation of Phylogenetic Dependence of Codon Usage

The 59 codons usage values for individual genome in each DENV dataset were tested for phylogenetic dependence. We followed Abouheif's Cmean approach. The null hypothesis of lacking phylogenetic dependence was rejected (*P* = 0.05) for all 59 codon variables with Abouheif's Cmean statistic for DENV1–3 (Table S5). In DENV4, the absence of phylogenetic autocorrelation was not significantly rejected for the following codons: CGT, CTG, TAC, TAT, TTC, TTG, and TTT. However, although the phylogenetic dependence varied across the 59 codons, the null hypothesis of lacking phylogenetic dependence was rejected for all DENV1–4 by means of Blomberg's *K* statistic (Table S6). The statistical results indicate the presence of phylogenetic dependence of codon usage in DENV genomes.

## 4. Discussion

The identification of principal factors shaping codon usage is important for understanding the evolution of organisms, including viruses. In the present study, the analysis of total GC relation with the three nucleotide positions of codons GC1, GC2, and GC3 showed that the forces shaping codon usage were not the same for all codon positions (Table S2). The GC3 had the highest correlation with total GC and was very close to the total GC value in DENV1–4, suggesting a strong mutational pressure on the third position of codons. The ENC or ENCp versus GC3 plots showed that, in addition to compositional constraint, some other factors have effect on the codon usage variations. GC2 does not have important correlation with total GC in the examined genomes in the present study, implying that the constraint on this codon position is possibly due to the functional selection. A recent paper showed that the mutations on this position in the analyzed samples were mostly nonsynonymous substitutions [15]. These results demonstrated that both mutational and purifying selection pressures are the major forces in influencing the codon usage among DENV, consistent with some previous reports [6, 38], but these factors have distinct pressure on specific nucleotide position of a codon.

The analysis of ENC showed an overall weak codon usage bias, as shown in Table S1, where DENV2 has the highest codon bias (48.80) and DENV4 has the lowest one (50.87). This result is similar to a recent report [14], indicating that the result was not affected by an increased number of samples and might represent an inherent feature of DENV. One plausible explanation could be that DENV4 is less adapted to human environment, whereas DENV2 is more adapted to humans. On the other hand, DENV2 has been associated with more aggressive diseases forms and is generally the most prevailing serotype during outbreaks situations [39]. These could mean that codon bias of DENV2 contributes to successful infection in human cells in comparison with DENV4.

Moreover, the CA and HCbT analyses within each serotype showed similar clustering patterns for the four serotypes. The DENV strains occurring in the same continental region are more closely related, forming a cluster, indicating that viruses from a geographical group show similar codon usage bias. The Asian genomes of the four serotypes showed a wide diversity in the clusters and each of them can be further divided into more homogenous subgroups. This more diversified clustering could be the consequence of longer times of DENV evolution in Asia than in other regions. Some of the Asian genomes clustered close to the American ones, implying an evolutionary link between the Asian and American clusters. The North and South American strains tend to cluster more homogenously together with less codon usage variations, corresponding to the previous observation that a limited nucleotide diversity exists in American DENV strains [1, 15]. As the DENV in North and South America came from Asia, the homogenous cluster in North and South American populations could indicate a simple event of introduction from Asia, then spreading over this continent with much less adaptation time than in Asia, as the consequence of founder effect.

The sequences isolated from mosquito and monkey genomes in the CA were also grouped with human strains from the same geographic origin, indicating that sylvatic DENV changes in adaptation on codon usage in a similar way to endemic human DENV, as indicated by the study on nucleotide sequences [40]. On the other hand, Zhou et al. reported no link of geographic origin to the codon usage of DENV [13]. Behura and Severson found that the silent sites are favoring the geographical diversification [15]. Our study showed that not only GC3 but also GC1 and GC2 have a good correlation with axis major variation, depending on the serotype, suggesting that all the codon sites are related to clustering of geographical strains. Thus, the present study demonstrated the strong influence of geographic origin of DENV on shaping codon usage patterns. The discrepancy in results from studies may be due to the magnitude of samples used for analysis.

The clustering groups based on the codon usage datasets or phylogenetic tree on nucleotide sequence dataset showed the similar clustering results. This observation indicates the influence of the species evolution of DENV at the level of codon usage. We applied two statistical methods to assess the phylogenetic dependence of codon usage values. The positive results suggested that codon usage of DENV is engaged in the evolution of DENV lineages. The phylogenetic dependence is often interpreted as an information provider on the evolutionary process or rate [32]. For instance, it is common to associate the lack of phylogenetic dependence with evolutionary lability and the presence of phylogenetic dependence with stabilizing selection. Thus, the phylogenetic dependence analysis in the present study suggests that stabilizing selection acts on codon bias.

In summary, the codon usage of DENV genomes was analyzed on a large scale. Our analysis demonstrated that both mutational and purifying selection pressures have important contribution to the codon usage; however, these factors have distinct pressure on specific codon nucleotide positions. The codon usage patterns of DENV genomes showed apparent geographic feature. The phylogenetic dependence analysis suggests that stabilizing selection acts on codon bias.

## Supplementary Material

The accession numbers in NCBI database of genomes tested were provided and the datasets of DENV1–4 genomes for codon usage analysis were shown in an individual excel file. In addition, some statistic results obtained from the analyses were presented in Tables S1–S6. The hierarchical clustering trees based on RSCU data and the phylogenetic trees based on the genome nucleotide sequences were shown in Figure S1.

## Figures and Tables

**Table 1:** The correlation analysis of GC, ENC, and ENCp with the first axis of major variation.

Serotype	A1∗ % of variation	GC(r)	GC1(r)	GC2(r)	GC3(r)	ENC(r)	ENCp(r)
DENV1	41.8	−0.40	0.87	−0.54	−0.88	−0.47	−0.18
DENV2	39.8	0.57	0.00	−0.79	0.78	0.19	−0.18
DENV3	40.9	−0.55	−0.55	0.61	−0.59	0.44	0.59
DENV4	25.4	−0.40	−0.37	0.66	−0.46	−0.55	−0.50

* represents axis 1 in the correspondence analysis.
