# Supplementary material for: Large-Scale Genomic Analysis of Codon Usage in Dengue Virus and Evaluation of Its Phylogenetic Dependence
Source: Biomed Res Int. 2014 Jul 17;2014:851425. doi: 10.1155/2014/851425 (PMC4124757; doi:10.1155/2014/851425)

Figure S1. Hierarchical clustering trees based on RSCU data and Phylogenetic trees based on the genome nucleotide sequences. All the clades in each analysis for DENV1-4 were marked in colors according to the geographic origin as red (African), green (Asian), Magenta (North American), Blue (South American), Orange (Oceanic). A-D) Hierarchical clustering trees based on RSCU values for DENV 1-4, respectively. E-H) Phylogenetic trees based on the GTR nucleotide substitution model for DENV 1-4, respectively.

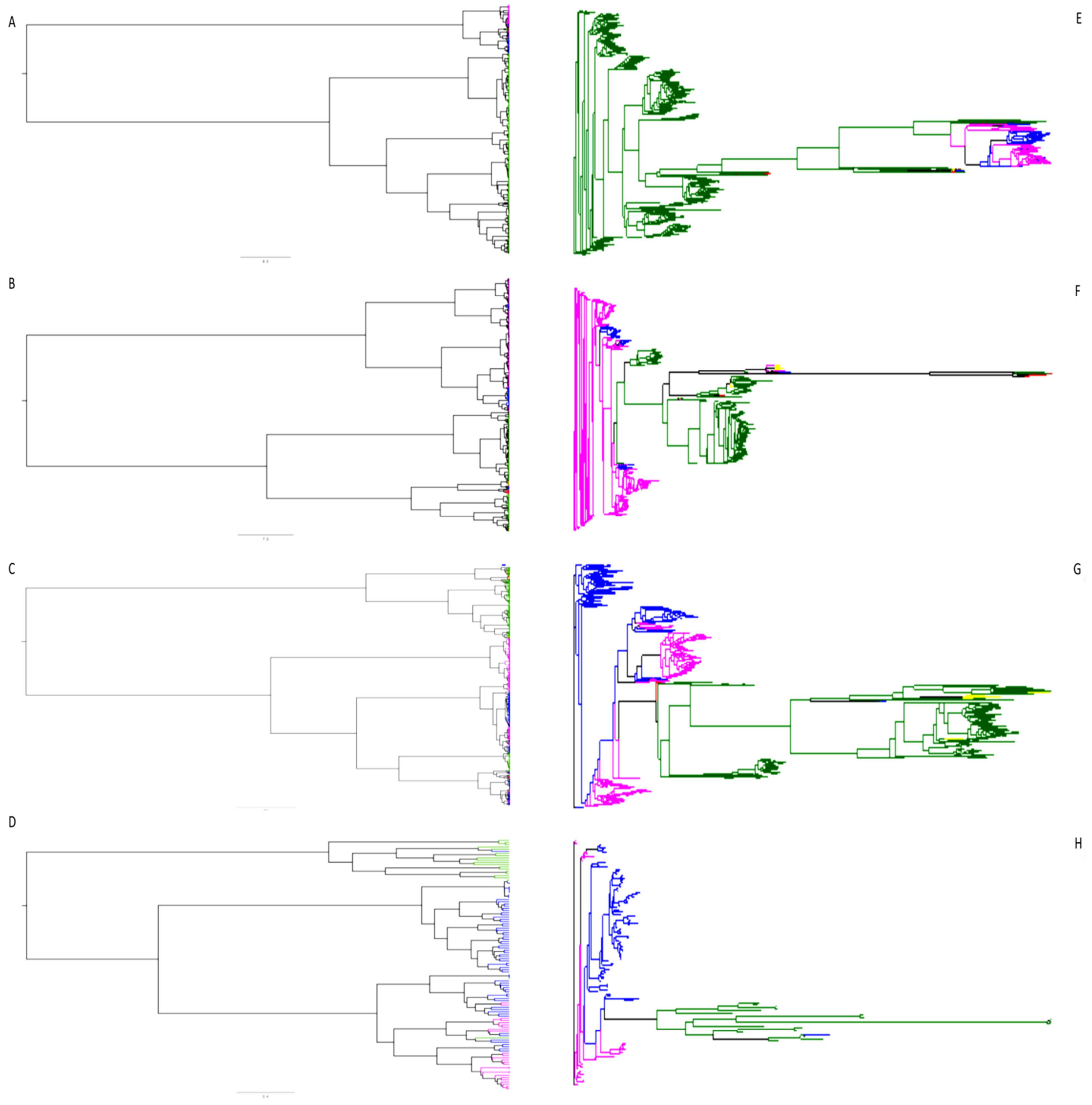

Supplement: Supplementary file 1 — The accession numbers in NCBI database of genomes tested were provided and the datasets of DENV1–4 genomes for codon usage analysis were shown in an individual excel file. In addition, some statistic results obtained from the analyses were presented in Tables S1–S6. The hierarchical clustering trees based on RSCU data and the phylogenetic trees based on the genome nucleotide sequences were shown in Figure S1. [file 851425.f1.zip › supplementary_materials/FigureS1.pdf]
